# Dynamical sensory representations establish a rapid odor code in a spiking model of the insect olfactory system

**DOI:** 10.1186/1471-2202-16-S1-P174

**Published:** 2015-12-18

**Authors:** Rinaldo Betkiewicz, Farzad Farkhooi, Martin Paul Nawrot

**Affiliations:** 1 Theoretical Neuroscience / Neuroinformatics, Freie Universität Berlin, Berlin, Germany; 2 Bernstein Center for Computational Neuroscience, Berlin, Germany; 3 Computational Systems Neuroscience, University of Cologne, Cologne, Germany

## 

In their natural environment, animals sense and evaluate olfactory cues of time-varying composition and concentration. Their olfactory pathways are adapted to the natural stimulus statistics, thus it is not surprising that odor processing is fast [1]. Honey bees, for example, learn to discriminate odors presented as short as 200 ms [2]. The neural odor code in these animals emerges within 50ms after stimulus onset and neural representation changes dynamically during and after an odorant is present [1,3]. How is the insect olfactory system optimized to reliably estimate spatial and temporal aspects of the olfactory environment and what are the mechanisms behind rapid odor processing?

To investigate odor encoding at the Antennal Lobe (AL) and the Mushroom Body (MB) level, we employ a simple phenomenological spiking network model of the honeybee olfactory system. The model implements a transformation from a low dimensional dense odorant representation in the AL to a high dimensional sparse representation in the MB. We demonstrate how information about the stimulus is present in both encoding schemes, by time resolved classification of neural activity.

**Figure 1:**
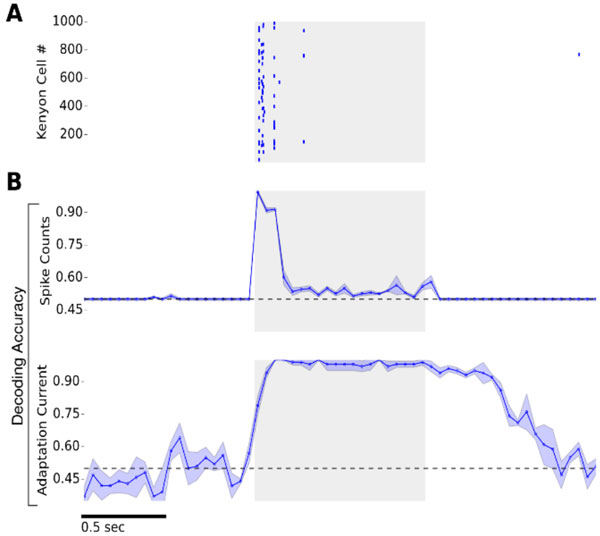
**(A) Kenyon Cell spike raster plot**. Stimulation is indicated by gray shading (**B**) Top: Decoding Accuracy given two odors (chance level: 0.5) as a function of time based on spike count estimates in 50ms time bins. Bottom: Decoding accuracy based on KC adaptation currents. Cellular adaptation levels provide a stable odor trace that persists as an odor afterimage.

Our model displays sparse and robust odor representation in the Mushroom Body [4]. Typically, less than 10% of the Kenyon Cell population is activated by an odor, with only 2-3 spikes at the odor onset (Figure 1A). KC spikes establish a rapid odor identity code at stimulus onset, while intrinsic adaptation currents provide a persistent and prolonged odor trace (Figure 1B). Our approach allows us to investigate dynamical changes in odor representations and predict odor after images.
